# A New 5α,8α-Epidioxysterol from the Soft Coral *Sinularia gaweli*

**DOI:** 10.3390/molecules18032895

**Published:** 2013-03-04

**Authors:** Wei-Hsuan Yen, Wu-Fu Chen, Ching-Hsiao Cheng, Chang-Feng Dai, Mei-Chin Lu, Jui-Hsin Su, Yin-Di Su, Yu-Hsin Chen, Yu-Chia Chang, Yung-Husan Chen, Jyh-Horng Sheu, Chan-Shing Lin, Zhi-Hong Wen, Ping-Jyun Sung

**Affiliations:** 1 Graduate Institute of Marine Biotechnology and Department of Life Science and Institute of Biotechnology, National Dong Hwa University, Pingtung 944, Taiwan; E-Mails: xyz78714@hotmail.com (W.-H.Y.); jinx6609@nmmba.gov.tw (M.-C.L.); x2219@nmmba.gov.tw (J.-H.S.); kb5634@yahoo.com.tw (Y.-H.C.); 2 National Museum of Marine Biology and Aquarium, Pingtung 944, Taiwan; E-Mails: gobetter04@yahoo.com.tw (Y.-D.S.); jay0404@gmail.com (Y.-C.C.); tony_chen72001@yahoo.com.tw (Y.-H.C.); 3 Department of Neurosurgery, Kaohsiung Chang Gung Memorial Hospital and Chang Gung University College of Medicine, Kaohsiung 833, Taiwan; E-Mails: ma4949@adm.cgmh.org.tw (W.-F.C.); ma4200@adm.cgmh.org.tw (C.-H.C.); 4 Institute of Oceanography, National Taiwan University, Taipei 112, Taiwan; E-Mail: corallab@ntu.edu.tw; 5 Department of Marine Biotechnology and Resources and Asia-Pacific Ocean Research Center, National Sun Yat-sen University, Kaohsiung 833, Taiwan; E-Mails: sheu@mail.nsysu.edu.tw (J.-H.S.); shinlin@mail.nsysu.edu.tw (C.-H.L.); 6 Doctoral Degree Program in Marine Biotechnology, National Sun Yat-sen University and Academia Sinica, Kaohsiung 804, Taiwan; 7 Graduate Institute of Natural Products, Kaohsiung Medical University, Kaohsiung 807, Taiwan

**Keywords:** *Sinularia*, epidioxysterol, cytotoxicity

## Abstract

A new sterol, (22*R*,23*R*,24*R*)-5α,8α-epidioxy-22,23-methylene-24-methyl-cholest-6,9(11)-dien-3β-ol (**1**), and two known sterols, (22*R*,23*R*,24*R*)-5α,8α-epidioxy-22,23-methylene-24-methylcholest-6-en-3β-ol (**2**) and 24-methylenecholestane-1α,3β,5α, 6β,11α-pentol (**3**), were isolated from the soft coral *Sinularia gaweli*. The structure of sterol **1** was established by spectroscopic methods and by comparison of the spectral data with those of known analogues. The cytotoxicity of sterols **1**–**3** towards various tumor cells is reported.

## 1. Introduction

**Figure 1:**
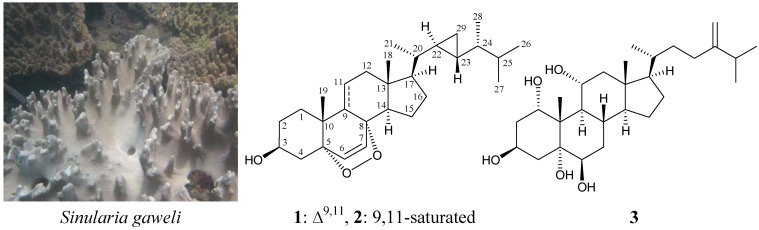
The soft coral *Sinularia gaweli* and the structures of (22*R*,23*R*,24*R*)-5α,8α-epidioxy-22,23-methylene-24-methylcholest-6,9(11)-dien-3β-ol (**1**), (22*R*,23*R*,24*R*)-5α,8α-epidioxy-22,23-methylene-24-methylcholest-6-en-3β-ol (**2**) and 24-methylenecholestane-1α,3β,5α,6β,11α-pentol (**3**).

Soft corals belonging to the genus *Sinularia* have been well-recognized as marine organisms containing various natural products that show interesting bioactivities [1,2,3]. A series of cytotoxic [4,5,6,7,8,9,10,11,12], anti-inflammatory [7,11,12,13] and antiviral [10] steroids have been isolated from *Sinularia* sp. octocorals collected off the waters of Taiwan. In continuation with our search for new natural substances, the organic extract of soft coral *Sinularia gaweli* (Figure 1) was studied, which displayed meaningful signals in NMR studies. Previous investigations of the chemical constituents of *S. gaweli* yielded two norcembranoidal diterpenes, 5-episinuleptolide acetate and scabrolide D [14]. In further studies of *S. gaweli*, a new sterol, (22*R*,23*R*,24*R*)-5α,8α-epidioxy-22,23-methylene-24-methylcholest-6,9(11)-dien-3β-ol (**1**), and two known sterols, (22*R*,23*R*,24*R*)-5α,8α-epidioxy-22,23-methylene-24-methylcholest-6-en-3β-ol (**2**) [4] and 24-methylenecholestane-1α,3β,5α,6β,11α-pentol (**3**) [15,16], were isolated (Figure 1). 

## 2. Results and Discussion

(22*R*,23*R*,24*R*)-5α,8α-Epidioxy-22,23-methylene-24-methylcholest-6,9(11)-dien-3β-ol (**1**) was isolated as a white powder. The molecular formula of **1** was established as C_29_H_44_O_3_ (eight degrees of unsaturation) from a [M+Na]^+^ molecule at *m/z* 463.3192 in HRESIMS (calcd for C_29_H_44_O_3_Na, 463.3188). The ^13^C-NMR and DEPT spectra of **1** showed this compound to have 29 carbons (Table 1), including six methyls, seven sp^3^ methylenes, eight sp^3^ methines, three sp^2^ methines, four sp^3^ quaternary carbons and an sp^2^ quaternary carbon. From the NMR spectra (Table 1), the presence of three oxygenated C atoms at *δ*_C_ 82.7 (C-5), 78.4 (C-8) and 66.3 (CH-3) in the ^13^C-NMR spectrum and an oxymethine proton at *δ*_H_ 4.02 (1H, m, H-3) in the ^1^H-NMR spectrum was determined. This sterol was further recognized as a 5α,8α-epidioxysterol by the presence of the characteristic signals for H-6 (*δ*_H_ 6.60, *J* = 8.0 Hz) and H-7 (*δ*_H_ 6.28, *J* = 8.0 Hz) in the ^1^H-NMR spectrum [4,17]. Four protons appeared at *δ*_H_ 0.14 (2H, m, H_2_-29), 0.33 (1H, m, H-23) and 0.55 (1H, m, H-22), indicating the presence of a cyclopropyl moiety in **1**. Two singlets, which appeared at *δ*_H_ 0.68 (3H) and 1.09 (3H), were attributed to Me-18 and Me-19, respectively. Four doublets at *δ*_H_ 0.91 (3H, *J* = 6.4 Hz), 0.86 (3H, *J* = 6.8 Hz), 0.89 (3H, *J* = 6.8 Hz) and 0.92 (3H, *J* = 6.4 Hz) were due to the presence of Me-21, Me-26, Me-27 and Me-28, respectively. The above data suggested that **1** is a peroxysteroid containing a 22,23-methylene-24-methyl moiety in the side chain. 

**Table 1:** ^1^H (400 MHz, CDCl_3_) and ^13^C (100 MHz, CDCl_3_) NMR data, ^1^H–^1^H COSY and HMBC correlations for sterol **1**.

Position	*δ*_H_ (*J* in Hz)	*δ*_C_, Mult.	^1^H–^1^H COSY	HMBC (H→C)
1	2.11 m; 1.70 m	32.6, CH_2_	H_2_-2	n.o.
2	1.91 m; 1.55 m	30.6, CH_2_	H_2_-1, H-3	C-3
3	4.02 m	66.3, CH	H_2_-2, H_2_-4	n.o.
4	2.14 dd (13.6, 2.0); 1.92 dd (13.6, 11.6)	36.1, CH_2_	H-3	C-2, -3, -5, -10
5		82.7, C		
6	6.60 d (8.0)	130.8, CH	H-7	C-4, -5, -8
7	6.28 d (8.0)	135.4, CH	H-6	C-5, -8, -9, -14
8		78.4, C		
9		142.5, C		
10		37.9, C		
11	5.42 dd (6.0, 2.0)	119.8, CH	H_2_-12	C-8, -10, -12, -13
12	2.28 dd (16.8, 6.0); 2.09 dd (16.8, 2.0)	41.2, CH_2_	H-11	C-9, -11, -13, -14, -17
13		44.1, C		
14	1.83 dd (12.0, 8.0)	47.8, CH	H_2_-15	C-12, -15
15	1.75 m; 1.61 m	21.2, CH_2_	H-14, H_2_-16	C-8, -13, -16
16	2.20 m	28.4, CH_2_	H_2_-15, H-17	n.o.
17	1.49 m	57.4, CH	H_2_-16, H-20	n.o.
18	0.68 s	12.6, CH_3_		C-12, -13, -14, -17
19	1.09 s	25.5, CH_3_		C-1, -5, -9, -10
20	0.88 m	39.7, CH	H-17, H_3_-21, H-22	C-17
21	0.91 d (6.4)	19.0, CH_3_	H-20	C-20, -22
22	0.56 m	24.2, CH	H-20, H-23, H_2_-29	n.o.
23	0.33 m	25.1, CH	H-22, H-24, H_2_-29	n.o.
24	0.55 m	44.9, CH	H-23, H-25, H_3_-28	n.o.
25	1.64 m	32.8, CH	H-24, H_3_-26, H_3_-27	C-24
26	0.86 d (6.8)	18.5, CH_3_	H-25	C-24, -25, -27
27	0.89 d (6.8)	20.7, CH_3_	H-25	C-24, -25, -26
28	0.92 d (6.4)	15.8, CH_3_	H-24	C-24, -25
29	0.14 m	10.5, CH_2_	H-22, H-23	C-20, -22, -24

**Figure 2:**
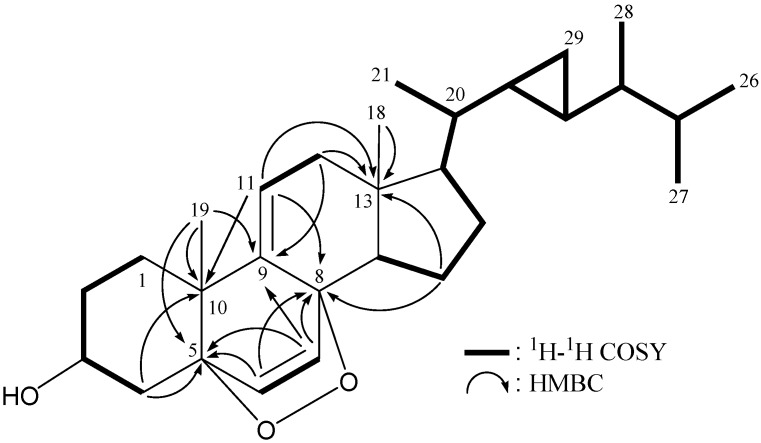
The ^1^H–^1^H COSY and selective HMBC correlations (protons→quaternary carbons) for sterol **1**.

From the ^1^H−^1^H COSY spectrum, several structural units, including H_2_-1/H_2_-2/H-3/H_2_-4, H-6/H-7, H-11/H_2_-12, H-14/H_2_-15/H_2_-16/H-17/H-20/H-22/H-23/H-24/H-25/H_3_-26(H_3_-27), H-20/H_3_-21, H-22/ H_2_-29, H-23/H_2_-29 and H-24/H_3_-28, were identified (Table 1 and Figure 2). These data, together with the key HMBC correlations between protons and quaternary carbons, such as H_2_-4, H-6, H-7, H_3_-19/C-5; H-6, H-7, H-11, H_2_-15/C-8; H-7, H_2_-12, H_3_-19/C-9; H_2_-4, H-11, H_3_-19/C-10; and H-11, H_2_-12, H_2_-15, H_3_-18/C-13, permitted the elucidation of the main carbon skeleton of 1(Table 1 and Figure 2). The ring junctions C-18 and C-19 methyl groups were positioned at C-13 and C-10 from the HMBC correlations between H_3_-18/C-12, -13, -14, -17 and H_3_-19/C-1, -5, -9, -10. An oxymethine unit at *δ*_C_ 66.3 correlated to the methine proton at *δ*_H_ 4.02 in the HMQC spectrum, proving the attachment of a hydroxy group at C-3.

**Figure 3:**
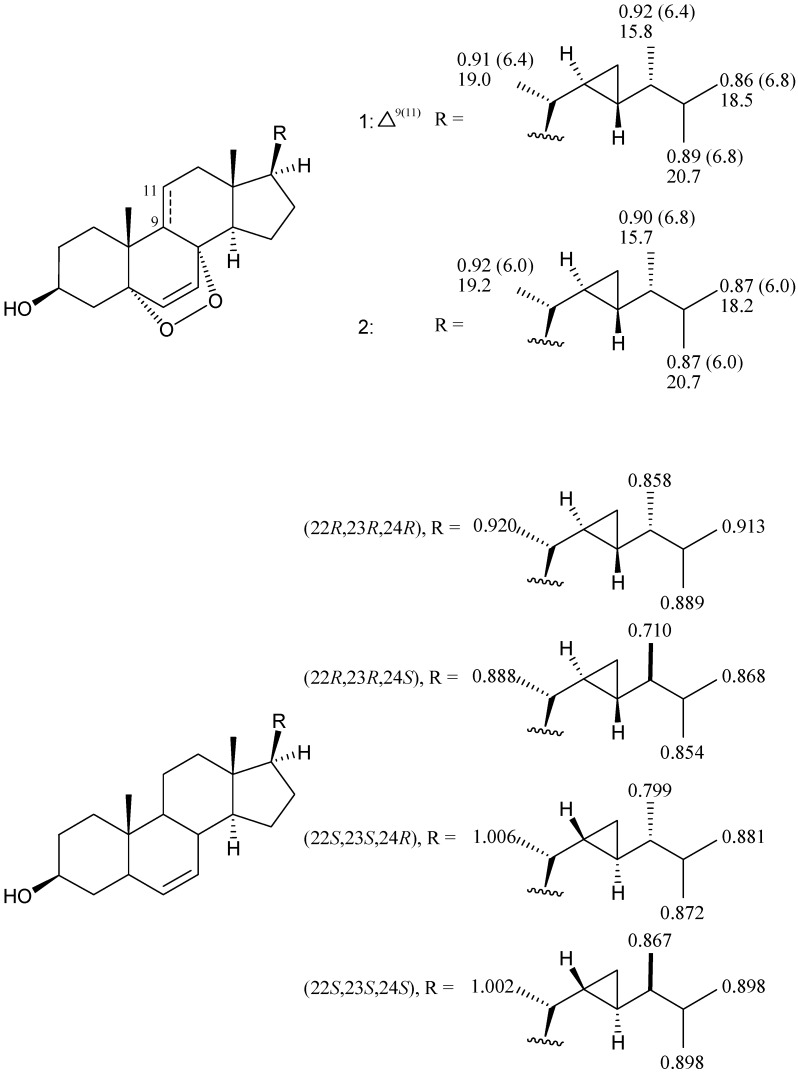
The ^1^H and ^13^C-NMR chemical shifts of the side-chain methyl groups of epidioxysterols **1** and **2** and synthetic isomers of demethylgorgosterols [4,18,19].

Because of the signals for protons H-22/H-24 and H-20/H_3_-21, H_3_-26, H_3_-27 are overlapped in the ^1^H spectrum of **1**, it is difficult to judge the relative configuration of the cyclopropyl moiety by their NOE effect in the NOESY spectrum. However, by comparison of the ^1^H- and ^13^C-NMR chemical shifts of Me-21, Me-26, Me-27 and Me-28 with those of a known epidioxysterol, (22*R*,23*R*,24*R*)-5α,8α-epidioxy-22,23-methylene-24-methylcholest-6-en-3β-ol (**2**) [4,18] and four synthetic demethyl-gorgosterol isomers [19] (Figure 3), it was suggested that the stereochemistry of **1** at the side chain should be assigned as 22*R*, 23*R* and 24*R*, as per those of **2**. The assignment of the carbon shifts of **1** was based on the comparison of these data with those of the tetracyclic system of **2** [4]. In the HMQC spectrum of **1**, the doublet methyls appearing at *δ*_H_ 0.86 (*J* = 6.8 Hz, H_3_-26) and 0.92 (*J* = 6.4 Hz, H_3_-28) showed ^1^*J*-correlations with *δ*_C_ 18.5 and 15.8, respectively; and the methine protons appearing at *δ*_H_ 0.33 (m, H-23) and 0.56 (m, H-22) showed ^1^*J*-correlations with *δ*_C_ 25.1 and 24.2, respectively. We suggest that the partial ^1^H and ^13^C-NMR chemical shifts for the side chain of steroid **2** that were reported previously should be re-examined [4,20]. Based on the above findings, the structure of **1** was tentatively established as (22*R*,23*R*,24*R*)-5α,8α-epidioxy-22,23-methylene-24-methylcholest-6,9 (11)-dien-3β-ol.

In previous studies, the 5α,8α-epidioxy sterols were supposed to have arisen from ∆^5,7^-sterols by photooxidization during storage and/or chromatographic separation [21,22,23] with a self-perpetuating mechanism [23]. ∆^5,7^-Sterol analogues were not obtained from *S. gaweli*; at this point it is difficult to infer whether epidioxysterol **1** from *S. gaweli* is a natural product or an artifact.

Sterols **2** and **3** were identified as (22*R*,23*R*,24*R*)-5α,8α-epidioxy-22,23-methylene-24-methylcholest-6-en-3β-ol and 24-methylenecholestane-1α,3β,5α,6β,11α-pentol, which have been previously isolated from a Formosan soft coral *Sinularia* sp. [4] and an Andaman Sea soft coral *Sinularia dissecta* [15,16], respectively. Their spectral data were in full agreement with those of previously reported. 

The cytotoxicity of sterols **1**–**3** towards K562 (human erythromyeloblastoid leukemia), MOLT-4 (human acute lymphoblastic leukemia) and HL-60 (human promyelocytic leukemia) cells was studied, and the results are shown in Table 2. These data showed that sterol **3** exhibited significant cytotoxicity towards HL-60 cells.

**Table 2:** Cytotoxic data of sterols **1**–**3**.

Compounds	Cell lines IC_50_ (μg/mL)		
	K562	MOLT-4	HL-60
**1**	NA	15.70	NA
**2**	NA	NA	12.14
**3**	9.71	6.91	3.39
Doxorubicin *^a^*	0.20	0.01	0.03

*^a^* Doxorubicin was used as the positive control. NA = not active at 20 μg/mL for 72 h.

## 3. Experimental

### 3.1. General Procedures

Optical rotation values were measured with a Jasco-P1010 digital polarimeter. Infrared spectra were obtained on a Varian Diglab FTS 1000 FT-IR spectrophotometer. NMR spectra were recorded on a Varian Mercury Plus 400 FT-NMR at 400 MHz for ^1^H and 100 MHz for ^13^C in CDCl_3_ or C_5_D_5_N at 25 °C. ESIMS and HRESIMS data were recorded on a Bruker APEX II mass spectrometer. Column chromatography was performed on silica gel (230–400 mesh, Merck, Darmstadt, Germany). TLC was carried out on precoated Kieselgel 60 F_254_ (0.25 mm, Merck) and spots were visualized by spraying with 10% H_2_SO_4_ solution followed by heating. Normal phase HPLC (NP-HPLC) was performed using a system comprised of a Hitachi L-7110 pump, a Hitachi L-7455 photodiode array detector and a Rheodyne 7725 injection port. A normal phase column (Supelco Ascentis^®^ Si Cat #:581515-U, 25 cm × 21.2 mm, 5 μm) was used for NP-HPLC. Reverse phase HPLC (RP-HPLC) was performed using a system comprised of a Hitachi L-7100 pump, a Hitachi L-2455 photodiode array detector and a Rheodyne 7725 injection port. A reverse phase column (Varian Polaris C18-A, 250 mm × 10 mm, 5 μm) was used for RP-HPLC.

### 3.2. Animal Material

Specimens of the soft coral *Sinularia gaweli* were collected by hand using scuba equipment off the coast of Sansiantai, Taitung County, Taiwan on Oct. 13, 2011, and stored in a freezer (−20 °C) until extraction. This organism was identified by comparison with previous descriptions [24]. A voucher specimen (NMMBA-TWSC-11007) was deposited in the National Museum of Marine Biology and Aquarium, Taiwan.

### 3.3. Extraction and Isolation

The freeze-dried and minced material of *Sinularia* *gaweli* (wet weight 1.30 kg, dry weight 328 g) was extracted with ethyl acetate (EtOAc) at 25 °C (2 L × 10). The EtOAc extract left after removal of the solvent (11.4 g) was separated by silica gel and eluted using *n*-hexane/EtOAc/acetone in a stepwise fashion to yield 14 fractions A–N. Fraction F was separated by NP-HPLC using a mixture of *n*-hexane and acetone (5:1) as the mobile phase to afford the subfractions F1–5. Subfraction F3 was further purified by RP-HPLC using a mixture of methanol (MeOH) and H_2_O (97:3, flow rate: 1.0 mL/min) to afford sterols **1** (0.5 mg, *t*_R_ = 40 m) and **2** (0.5 mg, *t*_R_ = 48 m). Fraction N was separated by NP-HPLC using a mixture of dichloromethane (CH_2_Cl_2_) and EtOAc as the mobile phase to afford the subfractions N1–10. Subfraction N9 was further purified by RP-HPLC using a mixture of MeOH and H_2_O (9:1, flow rate: 1.0 mL/min) to afford sterol **3** (1.2 mg, *t*_R_ = 31 m).

*(22R,23R,24R)-5α,8α-Epidioxy-22,23-methylene-24-methylcholest-6,9(11)-dien-3β-ol* (**1**): 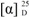 +158 (*c* 0.03, CHCl_3_); m.p. 218−220 °C; IR (neat) υ_max_ 3445, 1644 cm^−1^; ^1^H (400 MHz, CDCl_3_) and ^13^C (100 MHz, CDCl_3_) NMR data, see Table 1; ESIMS *m/z* 463 [M+Na]^+^; HRESIMS: *m/z* 463.3192 (calcd for C_29_H_44_O_3_Na, 463.3188).

*(22R,23R,24R)-5α,8α-Epidioxy-22,23-methylene-24-methylcholest-6-en-3β-ol* (**2**): 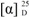 +20 (*c* 0.02, CHCl_3_) (Ref. [4], 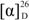 +35 (*c* 0.1, CHCl_3_)); IR (neat) υ_max_ 3438, 1638 cm^−1^; ^1^H (400 MHz, CDCl_3_) and ^13^C (100 MHz, CDCl_3_) NMR data were found to be in full agreement with those reported previously [4,18]; ESIMS *m/z* 465 [M+Na]^+^; HRESIMS: *m/z* 465.3347 (calcd for C_29_H_46_O_3_Na, 465.3344).

*24-Methylenecholestane-1α,3β,5α,6β,11α-pentol* (**3**): 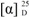 −3 (*c* 0.06, CHCl_3_) (Ref. [15], 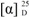 −4 (*c* 1.60, CHCl_3_)); IR (neat) υ_max_ 3380, 1216 cm^−1^; ^1^H (400 MHz, C_5_D_5_N) and ^13^C (100 MHz, C_5_D_5_N) NMR data were found to be in full agreement with those reported previously [15]; ESIMS: *m/z* 487 [M+Na]^+^; HRESIMS: *m/z* 487.3402 (calcd for C_28_H_48_O_5_Na, 487.3399).

### 3.4. Cytotoxicity Testing

The cytotoxicity of sterols **1**–**3** was assayed using a modification of the MTT [3-(4,5-dimethylthiazol-2-yl)-2,5-diphenyltetrazolium bromide] colorimetric method. Cytotoxicity assays were carried out according to previously described procedures [25,26].

## 4. Conclusions

Steroid metabolites are major constituents of the extracts of *Sinularia* spp. octocorals distributed in the waters off Taiwan [4,5,6,7,8,9,10,11,12,13]. Our studies on the chemical constituents of *Sinularia gaweli* have led to the isolation of a new epidioxysterol, (22*R*,23*R*,24*R*)-5α,8α-epidioxy-22,23-methylene-24-methyl- cholest-6,9(11)-dien-3β-ol (**1**), along with two known sterols, (22*R*,23*R*,24*R*)-5α,8α-epidioxy-22,23-methylene-24-methylcholest-6-en-3β-ol (**2**) and 24-methylenecholestane-1α,3β,5α,6β,11α-pentol (**3**). Sterol **3** was found to exhibit significant cytotoxicity against HL-60 tumor cells, and this result suggested that sterol **3** is worthy of further biomedical investigation. The soft coral *S.* *gaweli* has begun to be transplanted to culturing tanks with a flow-through sea water system located in the National Museum of Marine Biology and Aquarium, Taiwan for the extraction of additional natural products in order to establish a stable supply of bioactive material.
